# Giant cell-rich solitary fibrous tumour of the urinary bladder: case report of an unusual histological variant and literature review

**DOI:** 10.1186/s13000-024-01442-z

**Published:** 2024-01-23

**Authors:** Zhou Su, Jianguo Wei, Xiaolu Yuan

**Affiliations:** 1 Department of Pathology, Maoming People’s Hospital, Guangdong Medical University, Maoming City, China; 2 Department of Pathology, the First Affiliated Hospital of Zhengzhou University, Zhengzhou City, China

**Keywords:** Giant cell-rich solitary fibrous tumour, Urinary bladder, *NAB2-STAT6* gene fusion, Prognosis

## Abstract

**Background:**

Giant cell-rich solitary fibrous tumour (GCR-SFT), previously referred to as giant cell angiofibroma, is an uncommon soft tissue tumour that classically occurs in the orbit but very rarely presents in deep organs. Here, we present a case of GCR-SFT occurring in the urinary bladder, which is one of the unusual histological subtypes of SFT.

**Case presentation:**

A 56-year-old man was incidentally found to have a mass measuring 4.5 × 4.3 × 4.0 cm located in the left posterior wall of the bladder by computed tomography during a physical examination. The lesion was confirmed as GCR-SFT by pathological examination after laparoscopic radical surgery. Histopathologically, the tumour was a well-circumscribed, nonencapsulated lesion that was composed of bland spindle-ovoid tumour cells alternating with hypocellular and hypercellular areas, staghorn-like vasculatures and scattered large dark-stained multinucleate giant cells lining pseudovascular spaces. The spindle-ovoid cells and multinucleate giant cells showed strong and diffuse expression of CD34 and nuclear STAT6. In addition, the hallmark of the *NAB2ex4-STAT6ex5* fusion gene was detected by RT‒PCR. The patient was classified as having a low risk of recurrence or metastasis according to the risk stratification criteria. The patient underwent regular follow-up for 34 months after surgery, and there was no evidence of local recurrence or metastasis.

**Conclusion:**

This is the first reported case of GCR-SFT occurring in the urinary bladder with underlying *NAB2ex4-STAT6ex5* fusion. Complete surgical excision of the tumour and long-term follow-up are recommended to ensure no local recurrence or metastasis.

## Introduction

Solitary fibrous tumour (SFT) is an uncommon mesenchymal neoplasm of fibroblastic origin that usually involves the pleura and was first described by Klemperer and Rabin in 1931 [1]. Subsequently, it has been found that it can occur in numerous extrathoracic anatomical regions, such as the orbit [2], salivary glands [3], respiratory tract [4], mediastinum [5], adrenal glands [6], pelvis [7], skin [8], liver [9], and retroperitoneum [10]. However, SFTs occurring in the urinary bladder have seldom been reported.

Histopathologically, SFT characterized by *NAB2-STAT6* gene rearrangement mainly included “classic SFT” and “cellular SFT” previously recognized as haemangiopericytoma. Since giant cell angiofibroma (GCA), fat-forming haemangiopericytoma and the dedifferentiated type were essentially confirmed as SFT variants [11], the morphological spectrum of SFT had been greatly expanded, which posed great challenges to the diagnosis. However, the specific use of STAT6 immunohistochemistry and *NAB2-STAT6* gene detection by RT‒PCR make it possible to accurately diagnose SFT [12–15].

GCR-SFT, as a rare variant of SFT, has occasionally been reported to occur in the head and neck region, back, retroperitoneum, hip and vulva, etc., according to the literature [3, 10, 16]. However, to the best of our knowledge, there have been no reports of GCR-SFT occurring in the urinary bladder. It is not yet known whether tumour with different morphological features have different clinical courses. Hence, we detailed the clinical presentation, imaging examination, pathological features, immunophenotypes, molecular features and prognosis of the rare case in this study.

## Case presentation

**Fig. 1:**
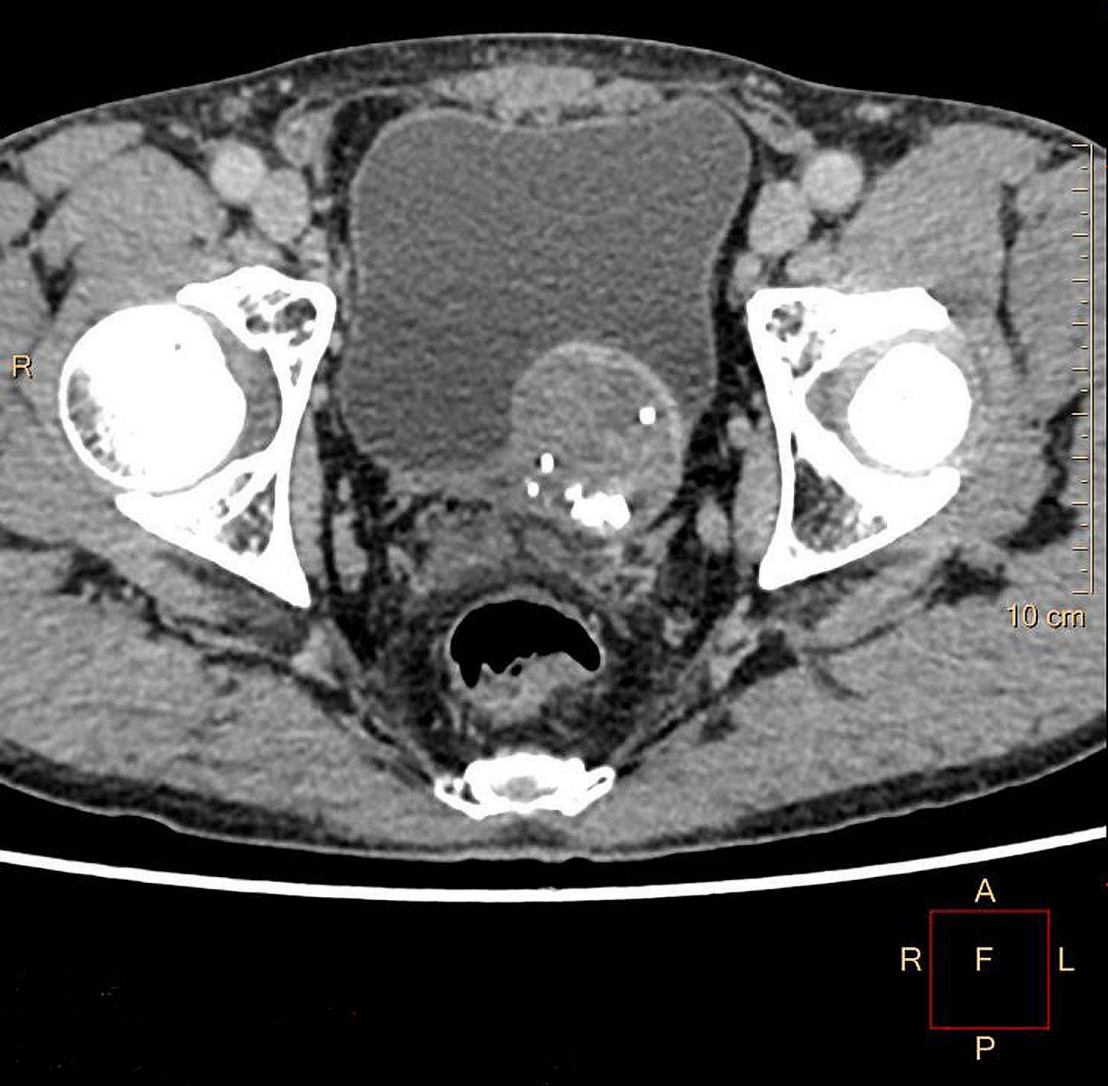
Contrast-enhanced axial CT revealed a 4.5 × 4.3 × 4.0-cm soft tissue mass located in the left posterior wall of the bladder with a clear boundary and uneven density protruding into the bladder cavity

A 56-year-old man presented to Maoming People’s Hospital for routine physical examination with no discomfort symptoms. Pelvic CT with contrast displayed a heterogeneously enhancing oval solid mass measuring 4.5 × 4.3 × 4.0 cm in the left posterior wall of the bladder (Fig. 1). The laboratory examination results showed that the urine occult blood test and urine protein were positive, while the liver function, renal function test and cancer markers were within normal limits. The patient underwent laparoscopic radical tumour resection.

**Fig. 2:**
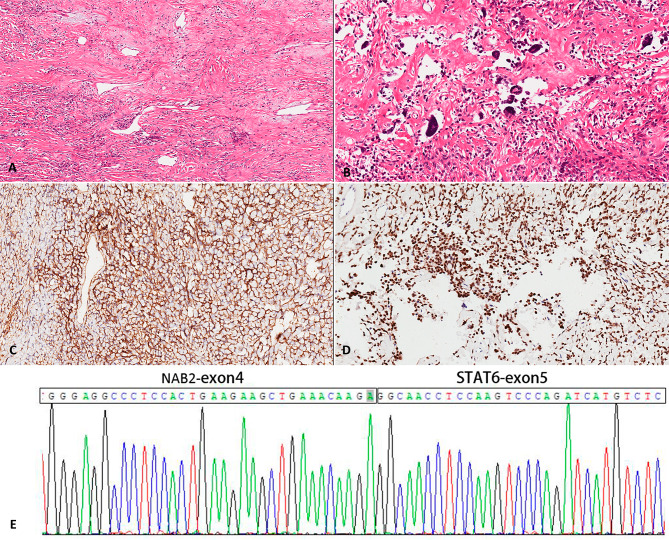
Histopathological features of GCR-SFT. (**A)** The tumour was characterized by the presence of spindle- or ovoid-shaped cells among sparse collagen fibres (H&E, magnification×40). (**B**) Vessels were dilated, staghorn-like appearance with remarkable scattered multinucleate giant cells lining pseudovascular spaces (H&E, magnification×200). The tumour cells were strongly positive for CD34 (**C**) and nuclear STAT6 (**D**) (immunohistochemistry, magnification×200). (**E**) The *NAB2ex4-STAT6ex5* fusion gene was revealed by RT‒PCR

Grossly, the tumour was a 5.0 × 4.0 × 4.0 cm in size with no obvious macroscopic haemorrhage or tumour necrosis on the cut surface. Histopathologically, the tumour was a well-circumscribed, nonencapsulated lesion that was composed of bland spindle-ovoid tumour cells alternating with hypocellular and hypercellular areas, staghorn-like vasculatures and scattered large dark-stained multinucleate giant cells lining pseudovascular spaces (Fig. 2A-B). There was no evidence of necrosis or mitotic activity (0/10HPF). Spindle-ovoid cells and multinucleate giant cells showed strong and diffuse expression of CD34 (Fig. 2C) and nuclear STAT6 (Fig. 2D), while being negative for S-100, Desmin, CD117, DOG-1, SMA, MDM2, P16, Pankeratin and CD68 (the antibody information is detailed in Table 1). The Ki-67 proliferation index of the tumour cells was 3%. In addition, RT‒PCR confirmed the presence of the *NAB2ex4-STAT6ex5* fusion gene in the tumour (Fig. 2E). Based on morphology, immunohistochemistry and molecular detection results, it was diagnosed as a GCR-SFT of the urinary bladder.

**Table 1:** List of immunohistochemical antibodies used in diagnosis

Antibody	Clone	Dilution	Source	Results
CD34	MX123	Ready-to-use	Maixin China	+++
STAT6	EP325	Ready-to-use	Maixin China	+++
S-100	4C4.9	Ready-to-use	Maixin China	-
Desmin	MX046	Ready-to-use	Maixin China	-
CD117	YR145	Ready-to-use	Maixin China	-
DOG-1	MX047	Ready-to-use	Maixin China	-
SMA	MX097	Ready-to-use	Maixin China	-
MDM2	1F2	Ready-to-use	Maixin China	-
P16	MX007	Ready-to-use	Maixin China	-
Pankeratin	CAM5.2	Ready-to-use	Maixin China	-
CD68	KP1	Ready-to-use	Maixin China	-
Ki-67	MX006	Ready-to-use	Maixin China	3%

+++, Strongly positive; -, Negative.

Based on the 3-tiered model (age at diagnosis, tumour size, mitotic count), the patient was given a score of 2 points (age ≥ 55 years and tumour size = 5 cm, shown in Table 2) and was stratified into low risk of metastasis or recurrence according to the risk assessment criteria of the 2020 WHO classification of soft tissue and bone tumours [17]. The patient underwent regular follow-up for 34 months after surgery, and there was no evidence of local recurrence or metastasis.

**Table 2:** Risk stratification model proposed by Demicco et al.

Risk Factor	Score	Our case
Age		
<55 years	0	
≥55 years	1	1
Tumour size		
<5 cm	0	
5-9.9 cm	1	1
10–15 cm	2	
Mitotic figure(/10HPF)		
0	0	0
1–3	1	
≥ 4	2	
Risk class	Total score	Total score
Low	0–2	2
Intermediate	3–4	
High	5–6	

## Discussion and conclusions

GCR-SFT, formerly known as GCA, was first reported in the orbital region by Dei Tos et al. in 1995 [18]. Since then, GCR-SFT has been described in some extraorbital anatomical locations, including the mediastinum, back, retroperitoneum, hip, vulva, and inguinal region [5, 16, 19]. In the most comprehensive review of the English literature to date, approximately 38 reports of GCR-SFT involving 66 cases have been identified between 1995 and 2023 [13, 16, 18–50]. However, GCR-SFT, as a rare variant, has never been reported to occur in the urinary bladder.

The clinical characteristics of the 66 reported cases of GCR-SFT are detailed in Table 3. Patients (Male38: Female28) ranged in age from 18 to 84 years with a mean age of 48.2 years. GCR-SFT mostly involved the orbit (*n* = 19, 28.8%) followed by the eyelid (*n* = 9, 13.6%), conjunctiva (*n* = 4, 6.1%), buccal mucosa (*n* = 4, 6.1%) and other head and neck regions (*n* = 17, 25.8%) including the parotid, occipital scalp, vocal cord, retroauricular region, submandibular, nasolacrimal duct, neck, tongue, parietal region, external auditory, nasopharynx, cheekbone, and sublingual regions. However, rare sites outside the head and neck region were noted in only 13 cases (back, groin, mediastinum, retroperitoneum, vulva, hip, axillary, gallbladder).

**Table 3:** Summarizing the 66 previous reported cases of GCR-SFT

Reference No./year	Gender/Age	Location/Size	CD34;STAT6 /RT-PCR^†^	Treatment	Follow-up (months)
1/1995	M/23	Eyelid/NA	+;ND/ND	Local excision	NSR(26)
	M/24	Eyelid/NA	+;ND/ND	Local excision	NSR(16)
	M/46	Eyelid/2 cm	+;ND/ND	Partial excision	Residual mass(14)
	M/27	Eyelid/NA	+;ND/ND	Local excision	Lost follow-up
	F/73	Orbit/ NA	+;ND/ND	Local excision	Local recurrence(60)
	M/59	Orbit/ NA	+;ND/ND	Local excision	Recent case
	M/73	Eyelid/ NA	+;ND/ND	Local excision	NSR(24)
2/1997	M/52	Orbit/1.2 cm	+;ND/ND	Lesion excision	NA
3/1998	F/62	Mediastinum/5 cm	+;ND/ND	Partial excision	NSR(8)
4/1999	F/78	Eyelid/2 cm	+;ND/ND	Biopsy	NA
	M/65	Orbit /NA	+;ND/ND	NA	NA
	M/47	Orbit/1.5 cm	+;ND/ND	NA	NA
	M/65	Conjunctiva	+;ND/ND	NA	NA
5/1999	F/49	Back/4 cm	+;ND/ND	Local excision	NSR(10)
6/1999	M/61	Buccal mucosa/1.5 cm	+;ND/ND	Simple tumorectomy	NSR(4)
7/1999	M/46	Eyelid/2 cm	+;ND/ND	Partial excision	NSR(24)
8/1999	F/46	Buccal mucosa/0.7 cm	+;ND/ND	Totally excision	NSR(6)
9/2000	F/55	Retroauricular region /2 cm	+;ND/ND	Simple tumorectomy	NSR(32)
	F/70	Orbit/1.3 cm	+;ND/ND	Simple tumorectomy	NSR(12)
	F/50	Back/3 cm	+;ND/ND	Simple tumorectomy	NSR(14)
	F/57	Back/NA	+;ND/ND	Simple tumorectomy	NSR(9)
	F/81	Occipital scalp/11 cm	+;ND/ND	Simple tumorectomy	NSR(7)
	F/36	Retroperitoneum/5 cm	+;ND/ND	Simple tumorectomy	NSR(24)
	F/18	Vulva/5.5 cm	+;ND/ND	Simple tumorectomy	Recent case
	M/34	Back/2.5 cm	+;ND/ND	Simple tumorectomy	Recent case
	M/41	Sub-mandibular Region/5 cm	+;ND/ND	Wide excision	NSR(17)
	M/33	Hip subcutaneous /4.5 cm	+;ND/ND	Wide excision	NSR(14)
10/2000	F/50	Inguinal region/10.8 cm	+;ND/ND	Simple tumorectomy	NSR(3)
11/2001	F/28	nasolacrimal duct region/2.2 cm	+;ND/ND	Wide excision	NSR(48)
12/2001	F/30	Soft tissue groin/ NA	+;ND/ND	Simple tumorectomy	NSR(31)
	F/37	Soft tissue groin/ NA	+;ND/ND	Simple tumorectomy	NSR(12)
	M/40	Parotid/ NA	+;ND/ND	Simple tumorectomy	NSR(52)
	F/41	Axillary soft tissue/ NA	+;ND/ND	Simple tumorectomy	NSR(4)
13/2004	M/60	Orbit/3 cm	ND;ND/ND	First: Partial excision; Second: totally excision	NSR(60)
14/2005	M/24	Conjunctiva/1.4 cm	+;ND/ND	Local excision	NSR(8)
15/2005	F/57	Eyelid/1.5 cm	+;ND/ND	Totally excision	NSR(12)
16/2006	M/43	Neck/14 cm	+;ND/ND	Surgical removal	NSR
17/2006	M/73	Orbit/1.5 cm	+;ND/ND	Totally excision (Second)	NSR
18/2006	M/68	Orbit/NA	+;ND/ND	Totally excision	NSR(6)
19/2007	F/83	Gallbladder/3.5 cm	+;ND/ND	Totally excision	NA
20/2008	M/44	Buccal mucosa/0.5 cm	+;ND/ND	Local excision	NSR(12)
21/2009	F/84	Tongue/2.5 cm	+;ND/ND	Local excision	NSR(8)
22/2009	F/16	Orbit/NA	+;ND/ND	Totally excision (Second)	NSR(20)
23/2010	F/25	Parotid/5.7 cm	+;ND/ND	Totally excision	NSR(24)
24/2010	M/40	Orbit/NA	+;ND/ND	Surgical debulking + radiotherapy	NSR(60)
25/2010	M/40	Vocal cord/1.2 cm	+;ND/ND	Local excision	NSR(12)
	M/45	Vocal cord/1 cm	+;ND/ND	Local excision	NSR(12)
26/2012	F/32	Occipital region of the scalp/2.4 cm	+;ND/ND	Totally excision	NA
27/2013	F/56	Eyelid/NA	+;ND/ND	Surgical debulking	NA
28/2013	F/30	Parietal region/6 cm	+;ND/ND	Totally excision	NSR(12)
29/2014	M/29	Parotid/5.8 cm	+;ND/ND	Totally excision	NSR(6)
30/2016	F/31	external auditory canal/1.8 cm	+; +/Neg	Totally excision	NA
31/2016	M/55	Nasal cavity/2.5 cm	-; +/3–19	Totally excision	NSR(7)
	M/47	Orbit/1.7	+; +/6–17	Totally excision	NSR(18)
	M/38	Orbit/2.3	+; +/6–17	Totally excision	Lost follow-up
	M/32	Orbit/1.8	+; +/ND	Totally excision	NSR(1)
	M/56	Orbit/4.2	+; +/ND	Totally excision	NSR(76)
	M/38	Orbit/3.4	+; +/6–17	Totally excision	NSR(1)
32/2018	M/65	Orbit/3.2 cm	+; ND/ND	Totally excision	NSR(24)
33/2020	M/64	Nasopharynx/3.9 cm	+; +/ND	Totally excision	NSR(14)
34/2020	M/84	Conjunctiva/1 cm	+; +/ND	Lesion excision	NSR(12)
	F/26	Conjunctiva/1 cm	+; +/ND	Lesion excision	NSR(6)
35/2020	M/57	Cheekbone/2 cm	+; +/ND	Totally excision	NA
36/2021	F/49	Sublingual region/3 cm	+; ND/ND	Surgical excision	NSR(6)
37/2022	M/47	Orbit/3.5 cm	+; +/3–18	Totally excision	NSR(3)
38/2023	M/47	Buccal mucosa/2 cm	+; ND/ND	Totally excision	NSR(1)

F, Female; M, male; NA, not available; ND: not done; Neg: negative result; NSR, no sign of recurrence; ^†^*NAB2–STAT6* fusion exon compositions

The clinical symptoms and signs depend on tumour size and location. The vast majority of bladder SFTs exhibit well-defined and slow-growing masses, with symptoms related to local compression caused by tumour growth, including urinary tract irritation, haematuria, difficulty urinating and lower abdominal discomfort [51–53]. The maximum diameter of GCR-SFT ranges from 0.5 to 14 cm with a mean size of 3.25 cm. Of note, tumours that occurred in areas such as the orbit and the eyelid often had a smaller diameter than tumours that occurred in subcutaneous soft tissue and deep organs. Although larger tumor diameters are positively correlated with higher risk stratification according to the risk assessment criteria, Feasel et al. demonstrated that 26 cases of SFTs occurring in cutaneous/subcutaneous soft tissue showed no recurrence or metastasis, and 2 cases of histologically malignant SFT were included [8].

Histopathologically, bland spindle-ovoid tumour cells alternating with hypocellular and hypercellular areas, staghorn-like vessels and prominent dark-stained multinucleate giant cells lining pseudovascular spaces are important diagnostic clues for GCR-SFT. For a long time in the past, a combination of CD34, BCL2 and CD99 has been widely used for the diagnosis of SFT. These markers often had good sensitivity and expression intensity in the vast majority of cases. Unfortunately, these markers are not specific and are frequently positively expressed in many spindle cell tumours closely mimicking SFT histologically [54, 55]. STAT6 has emerged as a useful tool for the diagnosis of SFT, and its sensitivity and specificity reached to 90–100% and 95–100%, respectively [13–15]. Based on the diagnostic needs, the use of CD34 and STAT6, which are better than other markers, is strongly recommended. However, subsequent research found that STAT6 can also be expressed in some other soft tissue neoplasms such as dedifferentiated liposarcoma (12%), desmoid fibromatosis (8%), and neurofibroma (8%) [56].

Given the unique or atypical morphology of cases, *NAB2-STAT6* fusion gene detection was performed to ensure accurate diagnosis. SFTs characteristically harbour inv12(q13q13)-derived *NAB2-STAT6* fusions with variable breakpoints resulting in diverse fusion variants. According to the literature, only 5 cases of GCR-SFT underwent *NAB2-STAT6* fusion gene detection, and fusion variants included *NAB2ex6-STAT6ex17* (3 cases), *NAB2ex3-STAT6ex18* (1 case) and *NAB2ex3-STAT6ex19* (1 case) [13, 50]. In this study, we first confirmed the presence of the *NAB2ex4-STAT6ex5* fusion gene in GCR-SFT of the urinary bladder. Nonetheless, the significance of such fusion variants in the GCR-SFT remains unclear. To date, no association has been found between a certain type of mutation variant and poor prognosis in SFT [13].

The differential diagnosis of GCR-SFT includes a number of soft tissue tumours, especially the so-called fibrohistiocytic tumours and fibroblastic/myofibroblastic tumours, such as deep benign fibrous histiocytoma and giant cell fibroblastoma. Deep benign fibrous histiocytoma, occurs mainly in the deep soft tissue or subcutaneous tissue and mostly presents as isolated, slow-growing nodules with branching haemangiopericytoma-like vessels. Furthermore, giant cell fibroblastoma is an intermediate soft tissue tumour that histologically overlaps with dermatofibrosarcoma protuberans.Giant cell fibroblastomas frequently occur in the subcutis and primarily affects children, although some adult cases have been reported. It consists of elongated wavy arrangements of spindle cells distributed in a mucinous-like or collagenous stroma, and multinucleate giant cells often lining larger lacunar or sinusoidal pseudovascular spaces that are irregularly distributed. However, STAT6 nuclear expression is absent in these tumours. A small number of other mesenchymal tumours express STAT6 including dedifferentiated liposarcoma, undifferentiated pleomorphic sarcoma, and nodular fasciitis [57]. However, their morphology is quite different from that of GCR-SFT.

In addition, the morphology of GCR-SFT may overlap with other giant cell-rich malignant bladder lesions including osteoclast-type giant cell-rich carcinoma and leiomyosarcoma with osteoclast-like multinucleated giant cells. Histologically, osteoclast-type giant cell-rich carcinoma showed biphasic morphology with polygonal to epithelioid to spindle mononuclear cells and scattered multinucleated osteoclast-like giant cells. Leiomyosarcoma with osteoclast-like multinucleated giant cells is composed of spindle cells and has the presence of numerous multinucleated, osteoclast-like giant cells. Immunohistochemically, in addition to expressing markers of their own intrinsic origin respectively, giant cells in both tumours expressed CD68 positively. However, the giant cells of GCR-SFT were negative for CD68.

Although GCR-SFT exhibits benign histological morphology and slow growth process, incomplete tumour resection may lead to recurrence after many years [29, 58]. Henceforth, complete surgical excision should be performed immediately after detection when eligible for surgery to ensure a positive outcome and minimize the chance of malignant transformation or metastasis [52]. For the management of SFT, extensive and healthy surgical margin resection is currently considered the gold standard.

The clinical course of SFTs can be predicted by establishing a risk stratification model for low, intermediate and high metastatic risk that takes into account age at diagnosis, tumour size, mitotic count, and necrosis [59, 60]. Our case was scored 2 points and classified as low-risk progression. The patient underwent a follow-up of 34 months after complete resection of the tumour and did not experience any local recurrence or metastasis. However, in light of the specific location of the tumour, long-term follow-up is still needed.

In summary, this is the first reported case of GCR-SFT occurring in the urinary bladder with underlying *NAB2ex4-STAT6ex5* fusion. Complete surgical excision of the tumour and long-term follow-up are recommended to ensure no local recurrence.

## Data Availability

The datasets used and/or analyzed during the current study available from the corresponding author on reasonable request.

## Abbreviations

SFT: Solitary fibrous tumour
GCA: Giant cell angiofibroma
STAT6: Signal transducer and activator of transcription 6
RT‒PCR: Reverse transcription-polymerase chain reaction
HPF: High power field
